# Effects of combined phytochemicals on skin tumorigenesis in SENCAR mice

**DOI:** 10.3892/ijo.2013.2005

**Published:** 2013-07-05

**Authors:** MAGDALENA C. KOWALCZYK, JACOB J. JUNCO, PIOTR KOWALCZYK, OLGA TOLSTYKH, MARGARET HANAUSEK, THOMAS J. SLAGA, ZBIGNIEW WALASZEK

**Affiliations:** 1 Department of Pharmacology, University of Texas Health Science Center at San Antonio, San Antonio, TX 78229, USA; 2 The Cancer Therapy and Research Center, University of Texas Health Science Center at San Antonio, San Antonio, TX 78229, USA; 3 Medical Research Division of the Regional Academic Health Center, University of Texas Health Science Center at San Antonio, San Antonio, TX 78229, USA

**Keywords:** skin, SENCAR mice, carcinogenesis, prevention, phytochemicals

## Abstract

The purpose of our study was to determine the effect of the combined action of phytochemicals on the early stages of skin tumorigenesis, i.e. initiation and promotion. We tested calcium D-glucarate (CG) given in the diet, while resveratrol (RES) and ursolic acid (UA) were applied topically. The 7,12-dimethylbenz[*a*]anthracene (DMBA)-initiated, 12-*O*-tetradecanoylphorbol-13-acetate (TPA)-promoted multistage skin carcinogenesis model in SENCAR mice was used. Mice received one topical dose of DMBA, then after one month, two weekly doses of TPA for 14 weeks until sacrifice. RES or UA were applied 20 min prior to DMBA or TPA treatment and 2% dietary CG was given from 2 weeks prior to 2 weeks after the DMBA dose or continually beginning 2 weeks prior to the first dose of TPA. UA applied alone and in combination with CG during the promotion stage was the only inhibitor of tumor multiplicity and tumor incidence. A number of combinations reduced epidermal proliferation, but only UA and the combination UA+CG applied during promotion significantly reduced epidermal hyperplasia. DMBA/TPA application resulted in significant increases in c-jun and p50, which were reversed by a number of different treatments. DMBA/TPA treatment also strongly increased mRNA levels of inflammation markers COX-2 and IL-6. All anti-promotion treatments caused a marked decrease in COX-2 and IL-6 expression compared to the DMBA/TPA control. These results show that UA is a potent inhibitor of skin tumor promotion and inflammatory signaling and it may be useful in the prevention of skin cancer and other epithelial cancers in humans.

## Introduction

The induction of cancer is a multistage process and its stages have been defined experimentally as initiation, promotion, and progression. Carcinogenesis depends on inherited and acquired susceptibility factors, such as mutations in oncogenes and tumor suppressor genes (1,2), on exposure to initiation factors, i.e. exogenous and endogenous carcinogens and on promotion and progression factors. It is well known that a variety of chemical and physical agents can cause skin cancer in rodents and man. Repetitive treatment with known skin carcinogens will lead to skin damage followed by inflammation and regenerative hyperplasia, dysplasia, papillomas, basal and/or squamous cell carcinomas (3–5).

Papilloma formation can be chemically induced by the two-stage carcinogenesis protocol (reviewed in ref. 6). Application of the carcinogen 7,12-dimethylbenz[*a*]anthracene (DMBA) to the skin surface results in *Ras* mutations in long-lived keratinocytes, probably stem cells (7,8). In response to applications of the tumor promoter 12-*O*-tetradecanoylphorbol-13-acetate (TPA), oncogenic transcription factors NFκB and AP-1 are activated (9–11), leading to increased inflammation, proliferation of initiated cells and finally to clonal expansion and papilloma formation. The mouse skin carcinogenesis model has provided great understanding of the important cellular and molecular events involved in tumor initiation, promotion and progression (3,4).

The mouse skin cancer model has provided an important system not only for studying mechanisms involved in the various stages of carcinogenesis and for bioassay of tumor-promoting and carcinogenetic agents, but also for the study of inhibitors of tumor formation and malignant conversion (5). This model has been used to show that a variety of naturally occurring phytochemicals may be very useful for the prevention of skin cancer as well as for the prevention of other epithelial cancers in humans (12). The mouse skin cancer model relates very well to other models where squamous cell carcinomas are induced. The phytochemicals may modify carcinogen activation, enhance phase II enzymes detoxification, modify antioxidant enzymes, prevent oxidative damage to DNA bases and mutations, decrease inflammation and proliferation, modulate the immune response and induce apoptosis. Because of their diverse mechanisms of action, many combinations of phytochemicals can interact in a synergistic fashion. One phytochemical may impact the metabolism of the other (13,14), or change its ability to enter or leave the cell (15). Phytochemicals may synergize by acting along different points on cell regulatory systems (16,17). Synergistic interactions between phytochemicals allow for a decreased concentration of each drug to achieve the same effect. This may reduce cost of the therapy as well as reduce potential side effects.

The phytochemicals selected for this study occur in many medicinal herbs and plants. Resveratrol (RES) is a naturally occurring phytoalexin associated with many health benefits, most notably the mitigation of age-related diseases, including neurodegeneration, carcinogenesis and atherosclerosis (18–20). RES has also shown promise as an antidiabetic (21) and anticancer (22,23) agent in recent clinical trials. Calcium D-glucarate (CG) is the salt and the commercial form of D-glucaric acid, which occurs naturally in a variety of foods, including broccoli, oranges and apples. Following oral administration, CG is converted to D-glucaro-1,4-lactone, which inhibits the enzyme β-glucuronidase and enhances phase II detoxification (24). Some *in vitro* and animal data suggest that inhibition of β-glucuronidase may suppress carcinogenesis (25), as well as inhibit the initiation and promotion/progression stages of tumorigenesis (12,26,27). Ursolic acid (UA) is a pentacyclic triterpenoid which has been shown to suppress skin (28) and breast (29) tumorigenesis. UA has also been found to induce apoptosis in a wide variety of cancer cells (30–32).

The overall goal of the present study was to determine the effect of combined action of phytochemicals on early stages of skin tumorigenesis, i.e. initiation and promotion. Our hypothesis was that concurrent topical and dietary treatment with selected compounds would lead to more efficient synergistic prevention of chemically-induced murine skin tumorigenesis. Tumors per mouse, tumor incidence, epidermal thickness, epidermal proliferation, and a number of inflammatory biomarkers were measured to determine the effects of these combinations of phytochemicals on the initiation and promotion stages of tumorigenesis.

## Materials and methods

### Scheme of DMBA-initiated, TPA-promoted skin carcinogenesis

Female SENCAR mice, 5 weeks old, were purchased from the National Cancer Institute, Frederick Cancer Research and Development Center (Frederick, MD, USA). At 6–7 weeks of age the backs of mice were shaved and 20 nmol of DMBA in 0.2 ml acetone (ACT) was applied topically, then after one month, two weekly doses of 2 μg TPA in 0.2 ml of ACT were applied for up to 14 weeks until sacrifice. The phytochemicals RES (2.5 μmol in 0.2 ml ACT) or UA (1 μmol in 0.2 ml DMSO) were applied topically to the dorsal surface of mice 20 min prior to DMBA or TPA treatment and 2% dietary CG was given in the AIN-93G diet from 2 weeks prior until 2 weeks after the DMBA dose or continually beginning 2 weeks prior to the first dose of TPA. The compounds were used in these same concentrations for the combination studies. After the 14th week of treatment, mice were euthanized, tumors were removed and two 1-cm^2^ skin samples were removed for histology and RNA extraction.

### Tumor counting

Upon the appearance of papillomas (7th week of TPA treatment) tumors on the backs of each mouse were counted weekly. Tumor multiplicity and tumor incidence were calculated for each group.

### Histological evaluation

The tissues were prepared for histological evaluation by using conventional paraffin sections and hematoxylin-eosin staining. The average epithelial thickness was determined from at least 12 randomly selected sites in formalin-fixed skin samples, with 3 measurements per image.

For proliferative analysis, mice were given an i.p. injection of BrdU (Sigma Chemical Co., St. Louis, MO, USA) (1.5 mg in saline per mouse) 60 min prior to sacrifice. The tissue sections were immunostained with anti-BrdU antibody (Lab Vision Corp., Fremont, CA, USA). The percentage of stained cells in the basal layer of the epidermis of 12 randomly selected sites was determined. For analysis of NFκB and AP-1 activities, slides were stained with anti-p50 (Lab Vision Co.) or anti-c-jun antibodies (BD Biosciences, Franklin Lakes, NJ, USA). Positive cells were counted as with BrdU samples, however, 3 mice per group were used in the analyses.

### Real-time PCR analysis

Total RNA was extracted using TRI reagent (MRC Inc., Cincinnati, OH, USA). RNA (1 mg) was reverse transcribed with oligo(dT), using cMaster RT kit (Eppendorf North America, Westbury, NY, USA) according to the manufacturer’s protocol. Primers used were: 5′-ATCCTGC CAGCTCCACCG-3′, 5′-TGGTCAAATCCTGTGCTCATA CAT-3′ for COX-2, 5′-GATGTGGAGCAACTTGGAAT-3′, 5′-AGCTCTCCACTTGCAGAAAA-3′ for IL-6 and 5′-CAT CCTGGCCTCGCTGTC-3′, 5′-CTCGTCGTACTCCTGC TTGGT-3′ for β-actin. Standard quantitative RT-PCR was performed in triplicate using SYBR Green RealMasterMix (Eppendorf North America) on the Realplex MasterCycler (Eppendorf). RT-PCR cycle thresholds (Ct) of candidate genes were normalized to control gene β-actin. The formula 2*Ct*(Candidate)/2Ct(Control) was used to calculate the normalized ratios.

### Statistical analysis

The results for Figs. 2–8 are expressed as means ± standard deviation (SD). For visual clarity, SD is not shown in Fig. 1. For comparison of the differences between the groups, a two-tailed, unpaired, Student’s t-test was used. p<0.05 was considered to be statistically significant.

## Results

### Effect of phytochemicals on tumor multiplicity and tumor incidence

**Figure 1:**
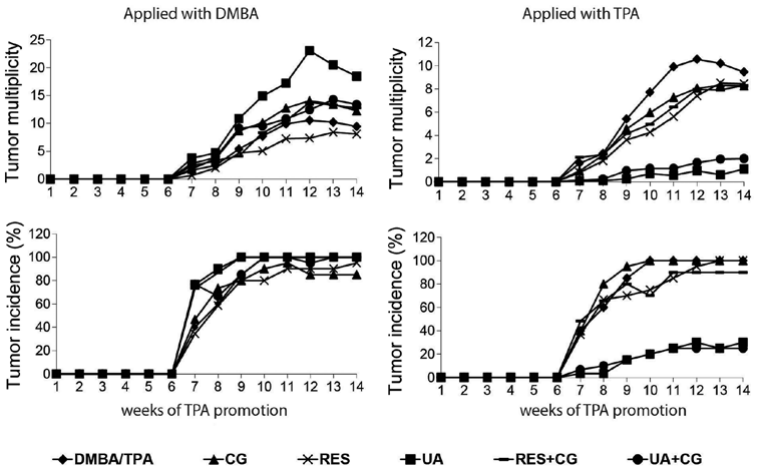
Effect of phytochemicals on DMBA/TPA-mediated tumor formation when applied with DMBA (left graphs) or TPA (right graphs).

Tumor multiplicity (average papillomas per mouse) and tumor incidence (% of mice with at least one papilloma) for each treatment group were determined by counting papillomas on the backs of mice once per week for the 7th through the 14th week of TPA treatment. No tested compound or combination inhibited carcinogenesis when applied during the initiation (DMBA) phase. In fact, UA significantly increased DMBA/TPA-mediated tumor formation when applied with DMBA in weeks 7 and 9–14 of TPA treatment (p<0.05). However, when applied with TPA, UA inhibited tumor multiplicity by 90% by itself and by 78.9% in CG-fed mice, and both treatments inhibited tumor incidence by 75% at the 14th week of TPA treatment. UA and UA+CG significantly p<0.05 inhibited tumor multiplicity when applied with TPA in each of weeks 7–14 of TPA treatment (Fig. 1).

### Effect of phytochemicals on epidermal hyperplasia and cell proliferation determined by incorporation of BrdU

**Figure 2:**
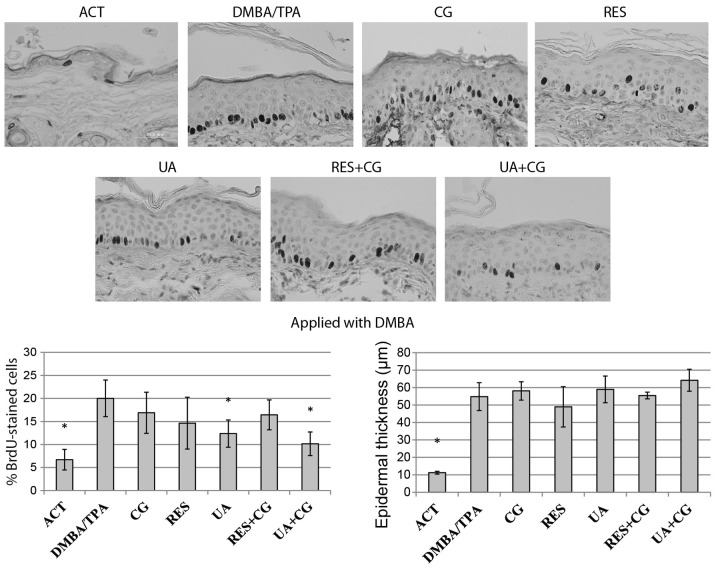
Effect of phytochemicals on DMBA/TPA-mediated epidermal proliferation and hyperplasia when applied with DMBA.

**Figure 3:**
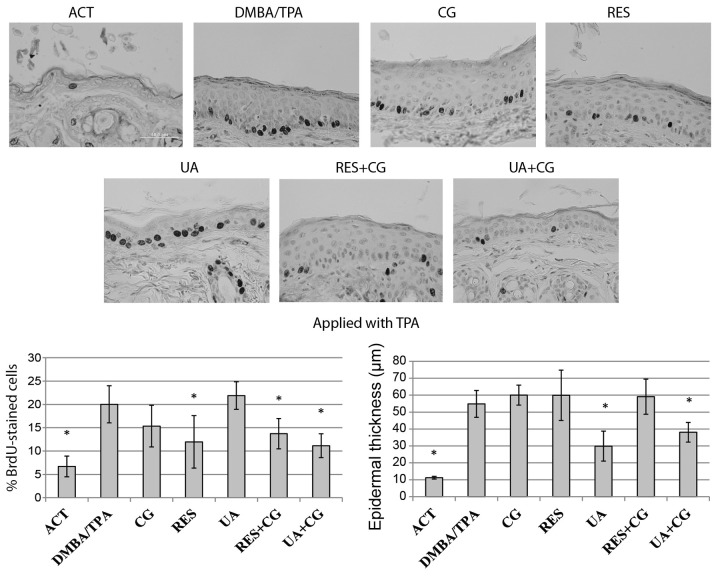
Effect of phytochemicals on DMBA/TPA-mediated epidermal proliferation and hyperplasia when applied with TPA.

The level of epidermal proliferation for each treatment group was determined by counting BrdU-positive cells at 12 random locations per sample, then expressing the data as a percentage of BrdU-positive cells in the basal layer. The proliferation rate of keratinocytes located in the basal layer of epidermis and overall epidermal thickness were greatly increased in the DMBA/TPA treated mice compared to negative control. Despite not having an effect on tumor formation when applied with DMBA, UA alone and in combination with CG was able to inhibit proliferation when applied during the initiation phase, while no compound significantly reduced epidermal hyperplasia when applied with DMBA (Fig. 2). All treatments except CG or UA alone had a statistically significant impact on proliferation when applied during promotion, however, only UA and UA+CG significantly affected epidermal hyperplasia when applied with TPA (Fig. 3). In similar studies, there is typically a strong concordance between levels of epidermal proliferation (measured by BrdU staining) and epidermal hyperplasia. However, in our experiments, we observed some disagreement between these two metrics of tumor promotion. For example, we observed that UA was the strongest inhibitor of epidermal hyperplasia when applied with TPA, however, it had no effect on epidermal proliferation (Fig. 3). This effect may be mediated by a UA-induced upregulation of apoptosis, which would decrease epidermal thickness without specifically affecting proliferation.

### Effect of phytochemicals on COX-2 and IL-6 expression

**Figure 4:**
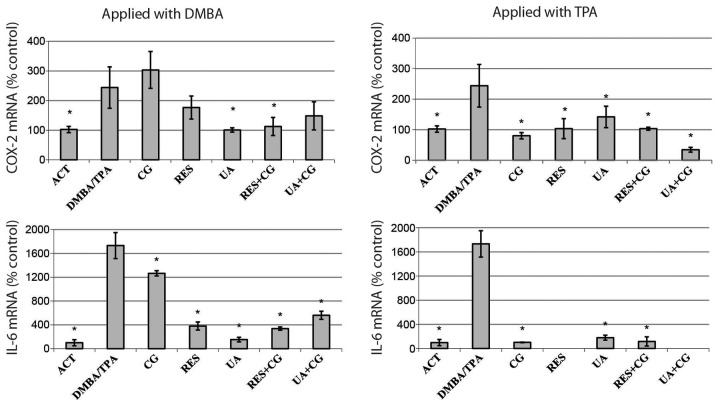
Effect of phytochemicals on COX-2 and Il-6 mRNA levels when applied with DMBA (left panels) or TPA (right panels).

DMBA/TPA treatments resulted in increased expression of the inflammation markers COX-2 and IL-6, compared to control. These genes play important roles in such inflammatory responses as edema formation and hyperplasia, as well as in papilloma development in mouse skin (33,34). While only UA and RES+CG significantly reversed COX-2 mRNA levels when applied with DMBA in the two-stage model, every compound or combination decreased COX-2 mRNA levels when applied alongside TPA during the promotion stage. All treatments strongly decreased IL-6 expression; moreover, anti-promotion treatments reduced IL-6 expression to the negative control level (Fig. 4).

### Effect of phytochemicals on c-jun-positive cells

**Figure 5:**
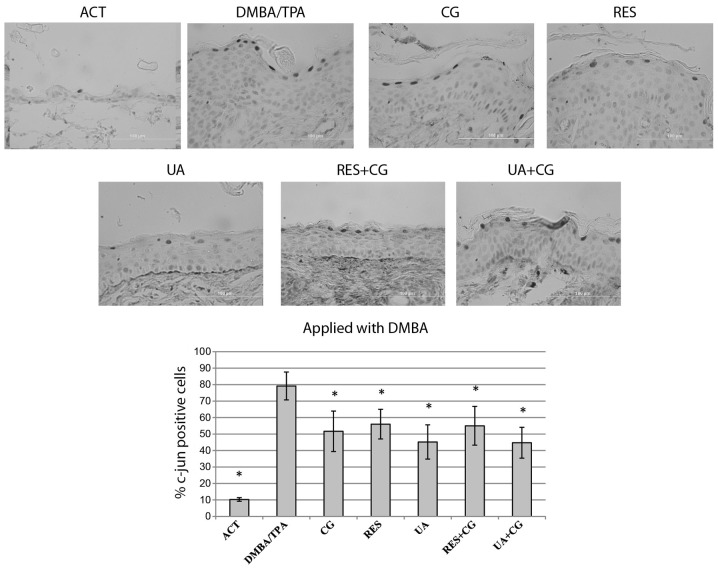
Effect of phytochemicals on c-jun positive cells when applied with DMBA.

**Figure 6:**
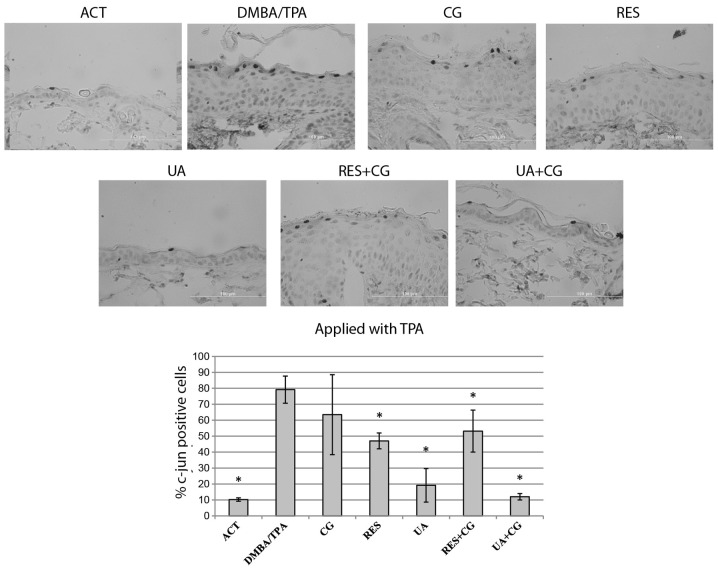
Effect of phytochemicals on c-jun positive cells when applied with TPA.

AP-1 activation levels were measured by immunostaining for c-jun-positive cells within the interfollicular epidermis. An average of 200 cells per tissue section were counted at 12 random locations per sample, with the data expressed as the mean ± SD of three animals per treatment group. As expected, DMBA/TPA treatment strongly increased the number of c-jun-positive cells. All treatments resulted in a slight decrease of c-jun-positive cells when applied with DMBA (Fig. 5). All treatments except for CG alone resulted in a significant decrease in c-jun-positive cells when applied alongside TPA. In case of mice treated with UA+CG during the TPA stage, the percentage of c-jun-positive cells was decreased to the negative control level revealing at least an additive effect of this combination (Fig. 6).

### Effect of phytochemicals on p50-positive cells

**Figure 7:**
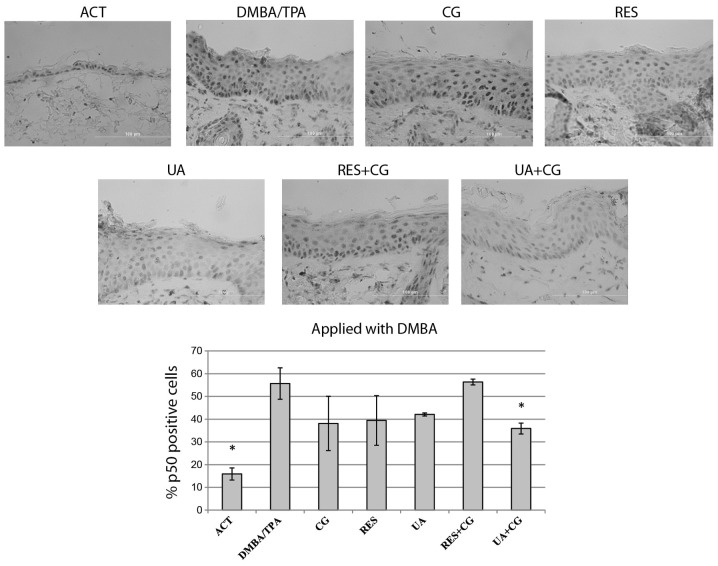
Effect of phytochemicals on p50 positive cells when applied with DMBA.

**Figure 8:**
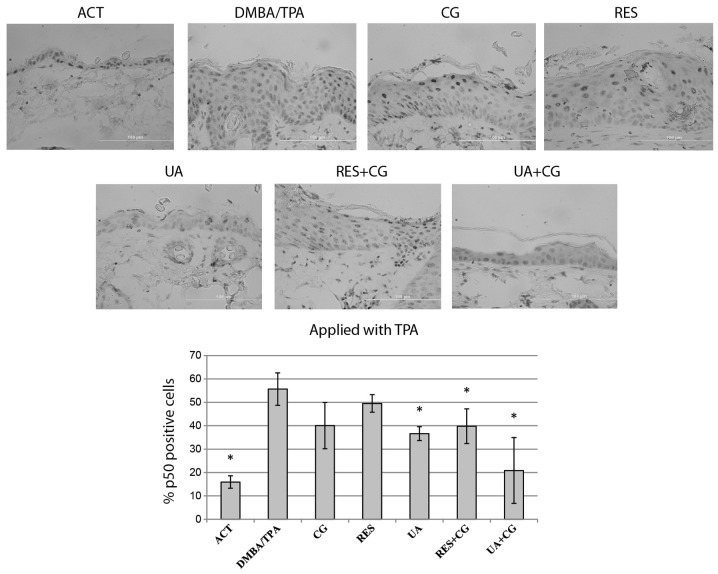
Effect of phytochemicals on p50 positive cells when applied with TPA.

NFκB expression for each treatment group was determined by counting p50 protein-positive cells at 12 random locations per sample, then expressing the data as a percentage of positive cells within the interfollicular epidermis. The DMBA/TPA-treated skin presented strong nuclear staining. UA+CG applied during the DMBA initiation stage was the only treatment to significantly suppress the percentage of p50-positive cells (Fig. 7). UA alone, its combination with CG and additionally dietary CG with RES during the promotion stage significantly reduced the percentage of p50-stained cells. UA+CG treatment applied during the promotion phase was the greatest inhibitor of the increase in p50-positive cells among used treatments, showing some additive effect in comparison to single treatments (Fig. 8).

## Discussion

DMBA/TPA treatments resulted in epidermal hyperplasia, characterized by a significant increase in cells positive for AP-1 and NFκB components with a simultaneously increased expression of the inflammation markers COX-2 and IL-6, compared to untreated controls. Despite our initial hypothesis, we observed no definitive synergistic effect between any of the tested phytochemicals.

UA applied alone and in combination with CG during the promotion stage was a strong inhibitor of tumor multiplicity and tumor incidence. While different treatments reduced epidermal proliferation when applied with either DMBA or TPA, only UA and the combination UA+CG applied during promotion significantly reduced hyperplasia. All anti-promotion treatments (either single compounds or their combinations), a majority of single treatments and all combinations during anti-initiation stage caused a marked decrease of inflammation related genes levels compared to the DMBA/TPA control. On the other hand, only UA+CG significantly diminished both c-jun-positive cells and p50-positive cells. These results show that UA alone or in combination with CG is a potent inhibitor of skin tumor promotion and it may be useful in the prevention of skin cancer and other epithelial cancers in humans. In addition, UA+CG is a strong inhibitor of AP-1 and NFκB, and may be useful for the prevention or treatment of maladies associated with activation of these factors.

In our study, we observed UA to be a much stronger inhibitor of tumor formation than RES or CG. This may be because UA and UA+CG were the most potent inhibitors of DMBA/TPA-induced c-jun positive cells and p50-positive cells of the treatments tested. This agrees with studies in other systems, which also show UA is a more potent inhibitor of inflammation-mediated processes such as monocyte recruitment than RES (35). Our other *in vivo* studies have demonstrated that UA more strongly inhibits TPA-induced phosphorylation and activation of NFκB subunit p65 *in vivo* (unpublished data). As AP-1 (36,37) and NFκB (9) activities have been shown to be necessary for chemically-induced skin cancer formation, we suggest that UA and UA+CG-mediated decreases in these transcription factors resulted in the observed antitumor effect.

None of the tested treatment groups significantly decreased tumor formation when applied during the DMBA stage. These compounds may only affect traits associated with skin tumor promotion, such as proliferation/clonal expansion, resistance to apoptosis and inflammation. In fact, UA added during the DMBA stage increased tumor formation. This may have been due to UA increasing the reactive concentration of DMBA. UA shows relatively little inhibition of CYP1A1 and CYP1B1, which metabolize polycyclic aromatic hydrocarbons like DMBA to their more reactive form (38). However, RES inhibits CYP1A1 and CYP1B1, but still showed no decrease in tumor multiplicity when applied with DMBA in our study. Another potential explanation is that the glucocorticoid-like structure of UA may cause an increase in nuclear envelope permeability (39), allowing for more DMBA intake and an increase in tumor formation. However, the classical glucocorticoid dexamethasone has been shown to inhibit skin tumor formation when applied with different initiators, including DMBA, in the two-stage skin carcinogenesis model (40). Finally, in our study, UA was applied topically in DMSO, as we could not solubilize the desired dose in ACT. Relative to ACT, DMSO has been shown previously to enhance solute penetration in skin explants of various animals (41) and enhance penetration of the carcinogen benzidine into porcine skin explants (42). The penetration-enhancing effects of DMSO may be mediated by a number of processes, including changing keratin structure and interactions with lipid bilayers (43), as well as the hygroscopic nature of DMSO (42). These effects of DMSO may have allowed for more DMBA epidermal penetration in the UA-treated mice in our study. However, we did not observe the same effect in the UA+CG group, which was also treated with UA in DMSO 20 min prior to DMBA application. Further studies are needed to determine how UA may increase chemically-mediated skin tumor formation when applied with DMBA.

Other studies have shown a potent antitumor effect of RES (44–46), including in the DMBA/TPA-mediated skin cancer model in Balb/c and ICR mouse strains (47,48). However, in our studies we observed no antitumor effect of RES, even at doses which significantly inhibited the transcription of inflammatory genes and the levels of c-jun protein. This lack of effect may be explained by the strain of mouse used. Our previous studies revealed RES has limited effect on epidermal hyperplasia and proliferation when applied with DMBA or TPA in short-term, DMBA- or TPA-only models in SENCAR mice (49) (unpublished data). However, in the same TPA-only model equimolar doses of UA reduced epidermal hyperplasia to the level of negative control. The lack of sensitivity to RES relative to UA observed in SENCAR mice may be explained by different levels of metabolizing enzymes or RES targets in the skin of different strains. Future studies, perhaps in a simplified system with keratinocytes isolated from each mouse strain, are needed to confirm this hypothesis.

These results indicate UA, alone or in combination with CG, functions as a potent preventer of chemically-induced skin cancer formation. Our previous experiments also identified combinations of natural compounds which synergistically inhibited DMBA-mediated mutation frequency as well as inflammation in the epidermis (49). Future studies may reveal these treatments to have an effect on full skin tumor formation or on other cancers.

## Abbreviations

RES: resveratrol
GC: calcium D-glucarate
UA: ursolic acid
DMBA: 7,12-dimethylbenz[*a*]anthracene
TPA: 12-*O*-tetradecanoylphorbol-13-acetate
